# Patterns of Nicotine Pouch Use Among Adults in the US, 2022-2023

**DOI:** 10.1001/jamanetworkopen.2025.31155

**Published:** 2025-09-08

**Authors:** Cristine D. Delnevo, Marisa Tomaino, Mary Hrywna, Michelle T. Bover Manderski

**Affiliations:** 1 Rutgers Institute for Nicotine & Tobacco Studies, New Brunswick, New Jersey

## Abstract

This cross-sectional study analyzes factors associated with nicotine pouch use among adults in the US.

## Introduction

Nicotine pouches are one of the only growing segments in a declining US tobacco market. When the US Food and Drug Administration authorized the first nicotine pouch in January 2025, it signaled that pouches may have population-level benefits, underscoring the need for study. Research points to low use among youths and adults overall,^1,2^ but suggests that nicotine pouches may appeal to users of other tobacco and nicotine products, including those interested in quitting.^1,2,3^ This study seeks to better understand nicotine pouch use among adults.

## Methods

This cross-sectional study was considered non–human participants research by Rutgers University and followed STROBE reporting guideline. The Tobacco Use Supplement to the Current Population Survey is a household-based survey fielded by the US Census every 3 years; detailed methods are found elsewhere.^4^ This analysis focused on the most recent wave (2022-2023) combining 3 months of data. Data were analyzed using the self-response weights to correct for varying probabilities of selection; variance was computed using replicate weights. Outcomes of interest were derived from 3 questions including ever use of nicotine pouches (yes or no), current use of nicotine pouches (daily, some days, or not at all), and number of days used in the past 30 days. Logistic regressions modeled the odds of ever, current, or daily use, with a 2-sided *P* < .05 indicating statistical significance; analysis was conducted using Stata version 18 (StataCorp). Covariates included demographics (sex, age, and race and ethnicity), census region, month of data collection, and cigarette, e-cigarette, and smokeless tobacco (SLT) use history. Self-reported race and ethnicity categories included non-Hispanic White and other (non-Hispanic American Indian and Alaska Native, non-Hispanic Asian, non-Hispanic Black, Hispanic, Native Hawaiian or Other non-Hispanic Pacific Islander, and multiracial) and were included given varying patterns of use of different tobacco products. We categorized individuals who quit a tobacco product by whether they quit before or after 2019^5^ to align with nicotine pouch availablity.^6^

## Results

The analytic sample included 110 084 adult self-respondents (50 560 male [weighted percentage, 48.51%]; 78 853 non-Hispanic White [weighted percentage, 61.37%]; 31 231 other race and ethnicity [weighted percentage, 38.63%]; mean [SD] age, 48.15 [10.06] years). The prevalence of ever (3199 respondents [weighted percentage, 2.65%]), current (484 respondents [weighted percentage, 0.42%]), and daily (229 respondents [weighted percentage, 0.18%]) use of nicotine pouches was low (Table 1). Demographic differences included significantly higher prevalence for adults who were male and non-Hispanic White. Ever, current, and daily use was virtually nonexistent for tobacco-naive adults. While higher prevalence was noted for adults with a history of tobacco use, current and daily use of nicotine pouches was highest among adults that recently quit another product.

**Table 1.** Nicotine Pouch Use Patterns Among US Adults by Demographics and Tobacco Use Status, 2022-2023 Tobacco Use Supplement^a^

Characteristic	Participants by tobacco use status, No. (weighted %) [95% CI] (N = 110 084)		
	Ever (n = 3199)	Current (n = 484)	Daily (n = 229)
Sex			
Male	2393 (4.24) [4.01-4.47]	425 (0.77) [0.67-0.88]	207 (0.34) [0.28-0.41]
Female	806 (1.15) [1.05-1.26]	59 (0.09) [0.06-0.12]	22 (0.03) [0.02-0.05]
Age group, y			
18-24	205 (2.48) [2.12-2.90]	56 (0.65) [0.47-0.91]	25 (0.25) [0.15-0.41]
25-44	1420 (3.54) [3.33-3.76]	273 (0.66) [0.56-0.77]	133 (0.30) [0.24-0.38]
45-64	996 (2.58) [2.38-2.79]	122 (0.30) [0.24-0.38]	56 (0.13) [0.10-0.17]
≥65^b^	578 (1.50) [1.36-1.66]	33 (0.10) [0.06-0.16]	15 (0.04) [0.02-0.07]
Race and ethnicity			
Non-Hispanic White	2748 (3.55) [3.38-3.73]	437 (0.58) [0.51-0.66]	213 (0.26) [0.22-0.32]
Other^c^	451 (1.21) [1.07-1.38]	47 (0.16) [0.11-0.23]	16 (0.04) [0.02-0.08]
Region			
Northeast	372 (2.03) [1.80-2.29]	50 (0.32) [0.23-0.44]	23 (0.16) [0.10-0.25]
Midwest	768 (3.30) [2.98-3.66]	125 (0.58) [0.47-0.71]	58 (0.26) [0.19-0.35]
South	1015 (2.38) [2.20-2.57]	119 (0.30) [0.24-0.38]	46 (0.10) [0.07-0.15]
West	1044 (2.98) [2.73-3.25]	190 (0.53) [0.42-0.68]	102 (0.25) [0.18-0.35]
Cigarette smoking status			
Daily^b^	748 (9.68) [8.84-10.59]	57 (0.81) [0.60-1.10]	14 (0.14) [0.07-0.26]
Some days^b^	208 (9.38) [7.94-11.05]	50 (2.07) [1.46-2.94]	20 (0.77) [0.41-1.45]
Quit since 2019	442 (13.57) [12.16-15.12]	66 (2.07) [1.52-2.80]	40 (1.41) [0.97-2.05]
Quit before 2019	1051 (5.53) [5.17-5.92]	119 (0.55) [0.44-0.69]	77 (0.32) [0.24-0.43]
Never	750 (0.90) [0.82-0.98]	192 (0.25) [0.21-0.30]	78 (0.09) [0.07-0.12]
E-cigarette use status			
Daily^b^	332 (17.86) [15.89-20.01]	45 (2.78) [1.78-4.29]	14 (0.75) [0.37-1.52]
Some days^b^	228 (16.52) [14.47-18.79]	43 (3.53)[2.40-5.18]	16 (1.05) [0.52-2.12]
Quit since 2019	531 (19.79) [17.89-21.84]	87 (3.08) [2.39-3.97]	41 (1.30) [0.86-1.96]
Quit before 2019	464 (16.86) [15.31-18.54]	51 (1.73) [1.22-2.47]	29 (0.91) [0.58-1.43]
Never	1644 (1.34) [1.25-1.44]	258 (0.22) [0.19-0.26]	129 (0.11) [0.09-0.13]
Smokeless tobacco use			
Ever	1850 (26.29) [24.98-27.65]	328 (4.73) [4.07-5.50]	153 (1.94) [1.60-2.35]
Never	1349 (1.18) [1.10-1.26]	156 (0.15) [0.12-0.18]	76 (0.07) [0.05-0.09]
Tobacco naive^d^			
Yes^b^	59 (0.09) [0.06-0.13]	20 (0.03) [0.02-0.05]	12 (0.02) [0.01-0.04]
No	3140 (7.16) [6.84-7.50]	464 (1.10) [0.97-1.25]	217 (0.46) [0.39-0.55]
Month and year of fielding			
September 2022	1130 (2.71) [2.51-2.92]	154 (0.34) [0.28-0.42]	75 (0.14) [0.11-0.19]
January 2023	1068 (2.78) [2.58-2.99]	160 (0.43) [0.35-0.53]	67 (0.15) [0.11-0.20]
May 2023	1001 (2.46) [2.27-2.66]	170 (0.48) [0.39-0.58]	87 (0.25) [0.18-0.33]
Overall	3199 (2.65) [2.52-2.78]	484 (0.42) [0.37-0.47]	229 (0.18) [0.15-0.21]

^a^ Prevalence estimates are weighted using self-response weights and the balanced repeated replication method.

^b^ Estimate may not be reliable for daily use of nicotine pouches, and caution should be used when interpreting.

^c^ Given very small cell sizes, non-Hispanic American Indian and Alaska Native (919 participants [weighted percentage, 0.69%]), non-Hispanic Asian (5360 participants [weighted percentage, 6.44%]), non-Hispanic Black (10 523 participants [weighted percentage, 12.08%]), Hispanic (12 643 participants [weighted percentage, 17.50%]), Native Hawaiian or Other non-Hispanic Pacific Islander [331 participants [weighted percentage, 0.29%]), and multiracial (weighted percentage, 1455 participants [1.63%]) participants were collapsed into the other race and ethnicity category.

^d^ Tobacco naive is defined as a response of no to ever using cigarettes, e-cigarettes, cigars, smokeless tobacco, hookah, pipe, or heated tobacco.

In multivariable analysis, increased odds of ever nicotine pouch use was observed for adults who were male (adjusted odds ratio [aOR], 1.66; 95% CI 1.47-1.88), living in the West (aOR, 1.56; 95% CI, 1.28-1.89), currently or formerly used SLT (aOR, 10.37; 95% CI, 9.11-11.79), and currently or formerly used cigarettes and e-cigarettes (ie, daily, somedays, quit since 2019, and quit before 2019); respondents who identified as another race or ethnicity had lower odds of ever nicotine use compared with White respondents (aOR, 0.55; 95% CI, 0.46-0.65) (Table 2). The associations with sex and race and ethnicity were larger when modeling current nicotine pouch use, and even larger when modeling daily use; odds of both outcomes increased over time. Current some days–smoking was associated with increased odds of current (cigarettes: aOR, 2.37; 95% CI, 1.39-4.04; e-cigarettes: aOR, 3.85; 95% CI, 2.26-6.57) and daily (cigarettes: aOR, 3.54; 95% CI, 1.67-7.50) pouch use; however, having quit cigarette smoking, especially since 2019, was significantly associated with daily pouch use (aOR, 3.89; 95% CI, 2.08-7.27).

**Table 2.** Adjusted Odds of Ever, Current, and Daily Nicotine Pouch Use, 2022-2023 Tobacco Use Supplement^a^

Characteristic	Ever use, aOR (95% CI)	*P* value	Current use, aOR (95% CI)	*P* value	Daily use, aOR (95% CI)	*P* value
Sex						
Female	1 [Reference]	NA	1 [Reference]	NA	1 [Reference]	NA
Male	1.66 (1.47-1.88)	<.001	3.63 (2.43-5.4)	<.001	4.99 (2.58-9.62)	<.001
Age group, y						
18-24	1 [Reference]	NA	1 [Reference]	NA	1 [Reference]	NA
25-44	0.87 (0.69-1.09)	.22	0.79 (0.54-1.17)	.24	0.76 (0.4-1.43)	.40
45-64	0.81 (0.63-1.03)	.09	0.46 (0.3-0.71)	.001	0.38 (0.19-0.75)	.01
≥65	0.61 (0.48-0.79)	<.001	0.22 (0.12-0.4)	<.001	0.13 (0.05-0.33)	<.001
Race and ethnicity						
Non-Hispanic White	1 [Reference]	NA	1 [Reference]	NA	1 [Reference]	NA
Other^b^	0.55 (0.46-0.65)	<.001	0.39 (0.26-0.61)	<.001	0.23 (0.12-0.47)	<.001
Region						
Northeast	1 [Reference]	NA	1 [Reference]	NA	1 [Reference]	NA
Midwest	1.14 (0.93-1.38)	.20	1.15 (0.78-1.7)	.46	1.12 (0.63-1.99)	.70
South	1.04 (0.86-1.24)	.71	0.85 (0.58-1.26)	.42	0.64 (0.36-1.14)	.13
West	1.56 (1.28-1.89)	<.001	1.60 (1.06-2.41)	.03	1.61 (0.90-2.90)	.11
Cigarette smoking status						
Daily	5.32 (4.51-6.27)	<.001	1.12 (0.74-1.71)	.59	0.68 (0.31-1.50)	.34
Some days	4.19 (3.20-5.50)	<.001	2.37 (1.39-4.04)	.002	3.54 (1.67-7.50)	.001
Quit since 2019	3.93 (3.18-4.86)	<.001	1.35 (0.85-2.14)	.20	3.89 (2.08-7.27)	<.001
Quit before 2019	3.06 (2.64-3.54)	<.001	0.94 (0.67-1.31)	.72	1.84 (1.15-2.92)	.01
Never	1 [Reference]	NA	1 [Reference]	NA	1 [Reference]	NA
E-cigarette status						
Daily	4.85 (3.93-5.99)	<.001	2.73 (1.46-5.08)	.002	0.93 (0.34-2.54)	.88
Some days	4.44 (3.53-5.59)	<.001	3.85 (2.26-6.57)	<.001	1.87 (0.79-4.45)	.15
Quit since 2019	4.96 (4.13-5.95)	<.001	2.78 (1.86-4.15)	<.001	1.77 (0.98-3.21)	.06
Quit before 2019	3.72 (3.10-4.47)	<.001	1.8 (1.16-2.8)	.01	1.59 (0.92-2.75)	.10
Never	1 [Reference]	NA	1 [Reference]	NA	1 [Reference]	NA
Smokeless tobacco use						
Ever	10.37 (9.11-11.79)	<.001	11.75 (8.22-16.79)	<.001	8.20 (5.17-13.01)	<.001
Never	1 [Reference]	NA	1 [Reference]	NA	1 [Reference]	NA
Month and year of fielding						
September 2022	1 [Reference]	NA	1 [Reference]	NA	1 [Reference]	NA
January 2023	1.07 (0.94-1.21)	.30	1.26 (0.93-1.71)	.13	0.97 (0.62-1.51)	.89
May 2023	1.01 (0.89-1.15)	.90	1.6 (1.18-2.17)	.003	1.85 (1.21-2.83)	.01

Abbreviations: aOR, adjusted odds ratio; NA, not applicable.

^a^ Adjusted for each variable included in the table.

^b^ Includes non-Hispanic American Indian and Alaska Native, non-Hispanic Asian, non-Hispanic Black, Hispanic, Native Hawaiian or non-Hispanic Pacific Islander, and multiracial.

## Discussion

This cross-sectional study, which, to our knowledge, presents the first estimates of daily nicotine pouch use in the US, highlights increased use over time and how use patterns differed by tobacco use status and demographic characteristics. Our findings add to a growing literature highlighting the appeal of nicotine pouches appeal to those who use other products.^1,2,3^ The association of nicotine pouch use with SLT use is expected given product similarities. However, to our knowledge, this is the first study to show that daily nicotine pouch use is most prevalent among adults who recently quit using another tobacco product, with the largest association, after SLT use, observed among adults who recently quit cigarettes. Importantly, very low nicotine pouch use was noted among those that quit before 2019, and future studies on the use of emerging products should consider the heterogeneity of former tobacco users. Limitations include the cross-sectional design, self-report survey data, and that some results should be interpreted with caution given small cell sizes. Continued surveillance is warranted to assess the population health impact of this rapidly growing segment of the tobacco marketplace.

## Notes

Supplement.
Data Sharing Statement
