# Supplementary material for: Patterns of Nicotine Pouch Use Among Adults in the US, 2022-2023
Source: JAMA Netw Open. 2025 Sep 8;8(9):e2531155. doi: 10.1001/jamanetworkopen.2025.31155 (PMC12418121; doi:10.1001/jamanetworkopen.2025.31155)
Supplement: Supplement. — Data Sharing Statement [file jamanetwopen-e2531155-s001.pdf]

Data Sharing Statement

Delnevo. Patterns of Nicotine Pouch Use Among Adults in the US, 2022-2023. *JAMA Netw Open*. Published September 08, 2025. doi:10.1001/jamanetworkopen.2025.31155

Data
Data available: No

Additional Information
Explanation for why data not available: We utilized publicly available data
